# Human AQP5 Plays a Role in the Progression of Chronic Myelogenous Leukemia (CML)

**DOI:** 10.1371/journal.pone.0002594

**Published:** 2008-07-09

**Authors:** Young Kwang Chae, Sung Koo Kang, Myoung Sook Kim, Janghee Woo, Juna Lee, Steven Chang, Dong-Wook Kim, Myungshin Kim, Seonyang Park, Inho Kim, Bhumsuk Keam, Jiyoung Rhee, Nam Hee Koo, Gyeongsin Park, Soo-Hyun Kim, Se-Eun Jang, Il-Young Kweon, David Sidransky, Chulso Moon

**Affiliations:** 1 Department of Otolaryngology–Head and Neck Surgery, School of Medicine, Johns Hopkins University, Baltimore, Maryland, United States of America; 2 Graduate Program in Human Genetics, School of Medicine, Johns Hopkins University, Baltimore, Maryland, United States of America; 3 Department of Internal Medicine, College of Medicine, The Catholic University of Korea, Seoul, Korea; 4 Department of Laboratory Medicine, College of Medicine, The Catholic University of Korea, Seoul, Korea; 5 Department of Internal Medicine, College of Medicine, Seoul National University, Seoul, Korea; 6 Department of Hospital Pathology, College of Medicine, The Catholic University of Korea, Seoul, Korea; 7 Department of Oncology, School of Medicine, Johns Hopkins University, Baltimore, Maryland, United States of America; Ordway Research Institute, United States of America

## Abstract

Aquaporins (AQPs) have previously been associated with increased expression in solid tumors. However, its expression in hematologic malignancies including CML has not been described yet. Here, we report the expression of AQP5 in CML cells by RT-PCR and immunohistochemistry. While normal bone marrow biopsy samples (n = 5) showed no expression of AQP5, 32% of CML patient samples (n = 41) demonstrated AQP5 expression. In addition, AQP5 expression level increased with the emergence of imatinib mesylate resistance in paired samples (p = 0.047). We have found that the overexpression of AQP5 in K562 cells resulted in increased cell proliferation. In addition, small interfering RNA (siRNA) targeting AQP5 reduced the cell proliferation rate in both K562 and LAMA84 CML cells. Moreover, by immunoblotting and flow cytometry, we show that phosphorylation of BCR-ABL1 is increased in AQP5-overexpressing CML cells and decreased in AQP5 siRNA-treated CML cells. Interestingly, caspase9 activity increased in AQP5 siRNA-treated cells. Finally, FISH showed no evidence of AQP5 gene amplification in CML from bone marrow. In summary, we report for the first time that AQP5 is overexpressed in CML cells and plays a role in promoting cell proliferation and inhibiting apoptosis. Furthermore, our findings may provide the basis for a novel CML therapy targeting AQP5.

## Introduction

Aquaporins (AQPs) are water channel proteins that facilitate transcellular water movements [1], [2]. Each of the ten known human AQPs has a unique expression pattern [1]. For example, AQP type 5 (AQP5) is found in various epitheliums including lacrimal [3], salivary [4], and airway submucosal glands [5], pancreatic epithelium [6], and type I pneumocytes [7]. Although the role of AQPs in human pathology has been explored extensively, only recently has its role in cancer become an area of interest. The ectopic expression of human AQPs has been reported to be frequently associated with various cancers. For instance, AQP1 is expressed in brain [8], colon [9], and pancreatic cancers [6] and the microvessels in multiple myeloma [10], AQP3 in renal cell carcinoma [11] and AQP5 in colon [9], pancreatic [6], and ovarian cancers [12]. We have also previously reported that AQP5 expression is associated with an early stage of colorectal cancer development^9^. Interestingly, the expression of human AQP1, AQP3, and AQP5 was detected in tumor-infiltrating lymphocytes surrounding bronchogenic cancer of lung [13]. AQP1, AQP3, and AQP5 are also simultaneously induced during lymphocyte activation [13].

In addition to the above expression studies, recent molecular and biochemical studies have alluded to the role of AQPs in human carcinogenesis. AQP1 is shown to play a role both in angiogenesis [14] and cell cycle control [9], [15], assisting cancer development. Our group has reported that the expression of wild-type AQP5 (AQP5) in a NIH3T3 mouse fibroblast cell line induced many phenotypic changes characteristic of transformation *in vitro* and *in vivo* by triggering signaling pathways activated through Ras, which is induced by phosphorylation of the PKA consensus site of AQP5 [16], [17]. Interestingly, athymic mice injected with NIH3T3 cells stably transfected with AQP5 exhibited a robust tumor formation [17]. Most recently, we have demonstrated that AQP5 promotes cell proliferation and that its overexpression is related with liver metastasis; we have also found molecular pathways based on the Ras/ERK/Rb signaling pathway as a mechanism to promote cell proliferation in colon cancer cells [18]. Furthermore, we have observed that AQP5 triggers invasion and epithelial-mesenchymal transition in BEAS-2B human bronchial epithelial cells; interestingly, AQP5 protein expression showed a significant association with earlier disease progression in NSCLC patients of more than 400 [19].

To build on our findings from the colon and lung cancer models, in this report, we have studied the AQP5 expression pattern and its role in human chronic myelogenous leukemia (CML). To date, no study has demonstrated the expression of AQP5 in hematologic malignancies. Here, we show that AQP5 is expressed in CML cells and that the expression level of AQP5 may be associated with the progression of CML. Furthermore, we hypothesize that, like in the solid tumors mentioned above, AQP5 may trigger cell proliferation in leukemic cells.

## Materials and Methods

### Cell lines and drugs

The human CML cancer cell lines EM-2 and LAMA-84 were purchased from DSMZ (Germany), K562 from ATCC (USA). Human monoblastic leukemia cell line U937 was also purchased from ATCC (USA). All cell lines were cultured in RPMI medium (Invitrogen) with 10% fetal bovine serum (Gibco) and antibiotics. All the cell lines were cultured at 37°C in a humidified environment containing 5% CO2. Imatinib mesylate was a kind gift from Novartis (Basel, Switzerland).

### Patient bone marrow samples

Human bone marrow biopsy and aspirate samples were obtained from randomly selected patents from St. Mary Hospital and Seoul National University Hospital (Seoul, Korea). 41 biopsy samples were from CML patients and 5 from normal bone marrow transplant donors. 41 aspirate samples were from the former and 3 from the latter. At the time of the blood donation, written informed consent to the participation in the research campaign was obtained. This study was approved by the institutional review boards of St. Mary Hospital and Seoul National University Hospital.

### RT-PCR analysis of AQP5 mRNA expression

We isolated RNA using Trizol (Invitrogen) and reverse-transcribed total RNA (8 µg) with Moloney Murine Leukemia Virus (M-MLV) reverse transcriptase (Invitrogen) and one hundredth of the DNA was used as a template for polymerase chain reaction (PCR). Reverse transcriptase polymerase chain reaction (RT-PCR) was performed at 30 cycles: 95°C for 1 min, 55°C for 1 min, and 72°C for 1 min. The expression levels of AQP5 were measured semiquantitatively in CML cell lines and normal bone marrow cells. (AQP5, sense: AAGAAGGAGGTGTGTTCAGTTGCCTTCTTCA and antisense: GTGTGCCGTCAGCTCGATGGTCTTCTTCCG; GAPDH, sense: GCCTCAAGATCAGCAAT and antisense: AGGTCCACCACTGACACGTT) For quantitative measurement of AQP5 mRNA in lung and breast cancer tissues, leukemia bone marrow sample, and peripheral blood (all from the tissue bank), QuantiGene® 2.0 RNA Quantification System using branched DNA technology (Sigma-Aldrich) was used according to manufacturer's instruction. For the calculation of the relative expression level of AQP5, the ratio between AQP5 mRNA and GAPDH mRNA was used.

### Immunohistochemistry

The expression of AQP5 in normal bone marrow (n = 5) and CML (n = 41) was examined by immunohistochemistry. Bronchial carcinoma tissues from The Catholic University of Korea tissue bank were analyzed simultaneously as positive control. Paraffin-embedded tissue sections, sliced in 4 µm thickness, were deparaffinized in xylene and were rehydrated through graded alcohol, and followed by endogenous peroxidase blocking with 0.3% hydrogen peroxide for 10 min. Antigen retrieval was performed using pressure cooker at 121°C, 10 Lb in sodium citrate buffer (pH 6.0) for 3 min. They were incubated with 10 µg/ml anti-AQP5 antibody (Calbiochem, EMD Chemicals Inc., Darmstadt, Germany) overnight at 4°C. After washing, the secondary antibody was applied and incubated for 30 min at room temperature. Finally, the slides were covered with the mixed solution of DAB and HRP substrate buffer to develop the color and washed with deionized water, and followed by hematoxylin counterstaining. The visualization procedure was performed using the DAKO REALTM EnVisionTM Detection System (DAKO, Glostrup, Denmark).

### Scoring of Immunohistochemistry

**Figure 1:**
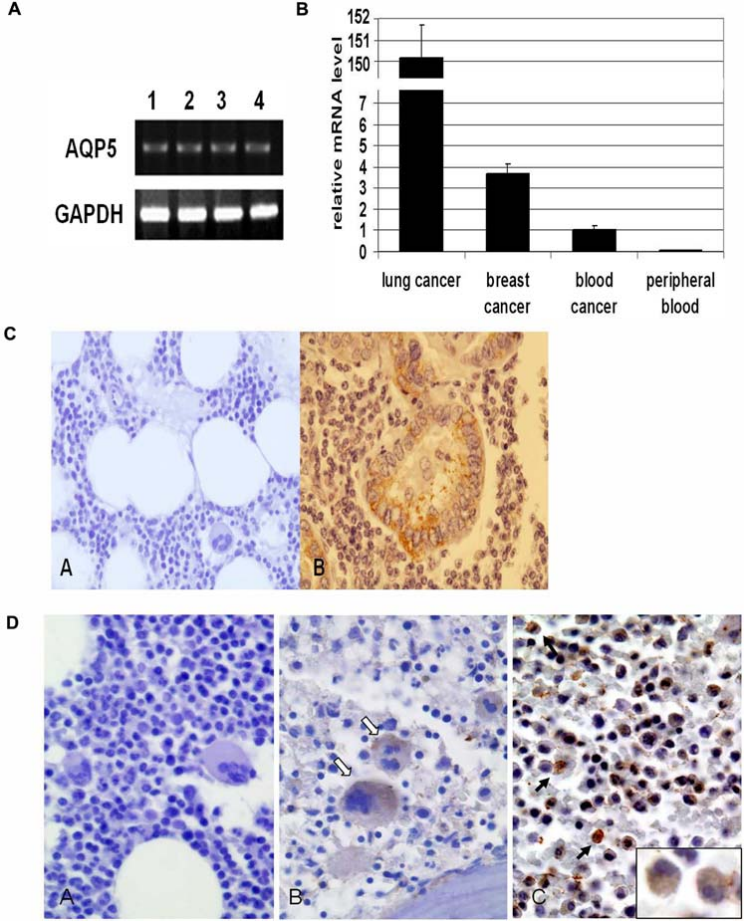
AQP5 expression assay. (A) RT-PCR analysis shows that AQP5 is expressed in human leukemic cell lines (1, K562; 2, U937; 3, EM-2; 4, LAMA-84). GAPDH is loaded as control. (B) The relative expression levels of AQP5, the ratio between AQP5 mRNA and GAPDH mRNA, from QuantiGene RNA Quantification System using branched DNA technology (Sigma-Aldrich) are compared between human lung cancer and breast cancer tissues, CML cells (blood cancer), and normal peripheral blood lymphocytes. While the first three showed expression of AQP5, AQP5 was rarely expressed in normal lymphocytes. (C) Immunohistochemical localization of AQP5 is shown in normal bone marrow (negative control) and lung adenocarcinoma (positive control). AQP5 immunostaining is negative for normal bone marrow (A, ×400), whereas AQP5 is localized in both apical and basal membrane of malignant lung tumor cells (B, ×400). (D) Photomicrographs of AQP5 immunostaining in CML bone marrow samples are shown with the examples of negative, trace(±), positive (+) immunoreactivity (×400). A; negative, B; trace(±), some megakaryocytes (open arrow) showing weak cytoplasmic staining, C; positive (+), granulocytes (arrow) showing strong staining and blasts (inlet) showing weak staining.

Immunostaining was graded semi-quantitatively considering staining intensity by two pathologists (M.K. and G.P.) blinded to the clinicopathologic variables. The staining intensity was arbitrarily scored on a scale of three grades: negative, trace, and positive (Figure 1A).

### Construction of hAQP5 cDNA expression construct

Human AQP5 cDNA was amplified by polymerase-chain reaction (PCR) using primers and then inserted into the EcoR I and XhoI sites of pcDNA 3.1(+). Cloned genes were confirmed by restriction analysis and by DNA sequencing of both strands. Expression constructs were transfected into K562 CML cell lines with Nucleofactor (Amaxa) according to manufacturer's recommendations. Transfection efficiency tested with enhanced green fluorescence proteins (Amaxa) was approximately 60–70 percent (Figure S1). Transfectants were selected with 600 µg/mL G418 for K562 cell line.

### Immunoblotting

Lysates from cultured cells and tissues were prepared in ice-cold NP-40 lysis buffer (10 mM Tris-Cl (pH 7.4), 137 mM NaCl, 10% glycerol and 0.1% Nonidet P-40) containing an inhibitor cocktail of 10 mM β-glycerol phosphate, 1 mM phenylmethylsulfonyl fluoride, 10 mM NaF, 10 mM Na orthovanadate, 4.5 U/ml aprotinin (Sigma), and 1 µg/ml leupeptin (Sigma). Crude protein lysates (25 µg) were separated by 4–12% SDS-PAGE (Invitrogen), transferred to nitrocellulose membranes (BIORAD), and blocked for 1 hour with 5% nonfat dry milk in Tris-buffered saline with 0.05% Tween-20. The following commercial antibodies were used for Western blot analysis: anti-AQP5 (1∶200; Alpha Diagnostic), anti-phospho-Bcr (1∶1000; Cell Signal), anti-c-Abl (1∶1000; Cell Signal), anti-phospho-Akt (1∶1000; Cell Signal), and anti-beta actin antibody (1∶5000; Sigma). Appropriate anti-rabbit and anti-mouse horseradish peroxidase-conjugated secondary antibodies (1∶5000; Amersham) were used. Immunoreactive bands were detected by enhanced chemiluminescence (Pierce).

### Small interfering RNA (siRNA) treatment

One pool containing four siRNAs targeting human AQP5 and a second pool containing four nonspecific siRNAs, as a negative control, were designed and synthesized to have the least off-target effects using SMART-pooled technology (Dharmacon). All siRNA sequences are described in the Supplementary Methods S1. Cells were transfected with 100 nM of siRNAs in Opti-MEM I reduced serum medium (Invitrogen) using RNAi Max transfection reagent (Invitrogen) according to the manufacturer's instruction and cultured at 37°C in a 5% CO_2_ atmosphere. The medium was removed and replaced with fresh RPMI culture medium supplemented with 10% FBS. The growth of each cell line was measured by MTT assay three days after treatment. Measurements were made in triplicate for each of the cell lines and the experiments were repeated three times.

### Cell proliferation assay

WST-1 assay were used to evaluate cell proliferation rate (Cell Counting Kit-8, Dojindo) in both cDNA and siRNA transfection experiements. ATP concentration was measured (CellTiter-Glo® Luminescent Cell Viability Assay, Promega) to assess cell viability in siRNA experiments. For cDNA transfection experiements, cells were grown in six-well plates at a density of 2.5×10^5^ cells per well for 4 days. For siRNA experiments, cells were cultured in six-well plates at a density of 5×10^5^ cells per well for 3 days. Measurements were made in triplicate for each of the cell lines and the experiments were repeated three times

### Apoptosis assay

Caspase 3, 8, and 9 concentrations were measured (Caspase-Glo® Luminescent Assay, Promega) to assess apoptosis activity in siRNA experiments. Cells were grown in six-well plates at a density of 5×10^5^ cells per well for 3 days. Light microscopy and Trypsin Blue staining was used to describe cells undergoing apoptosis. Measurements were made in triplicate for each of the cell lines and the experiments were repeated three times.

### Flow cytometry analysis

Flow cytometry was performed to evaluate BCR-ABL1 phosphorylation in AQP5/control siRNA and imatinib-treated (positive control) LAMA-84 CML cell lines. First, cells were washed in cold PBS twice, fixed with 2% formaldehyde for 10 min at 37°C, permeabilzed with ice-cold 90% methanol for 30 min, and aliquot 5×10^5^ cells into each assay tube. Then, cells were washed and resuspended with incubation buffer (0.5% bovine serum albumin in PBS) and incubated for 10 min. Rabbit anti-phospho-Bcr antibody (1∶50; Cell Signal) was added to the tube for 1 hr incubation. After washing and resuspension with incubation buffer, FITC-conjugated donkey anti-rabbit antibody (Jackson Immunoresearch) was applied for 30 min incubation. Finally, cells were rinsed with incubation buffer and resupended in 0.5 ml PBS. Stained cells were analyzed on a FACS Caliber (Becton Dickinson, San Jose, CA) within 30 minutes, and FACS data were analyzed using Cell Quest software (Becton Dickinson).

### Cytogenetic response analysis

A conventional metaphase karyotyping was performed with each bone marrow examination. Bone marrow aspirate was introduced into the culture medium, and harvested at 48 hours. The harvest and slide preparations were completed according to standard methods and G-banded metaphases were analyzed. BCR/ABL amplification was also confirmed using interphase fluorescence in situ hybridization (FISH) using LSI BCR/ABL dual color, dual fusion translocation probe (Vysis Inc., Des Plaines, IL). Furthermore, bcr/abl mutation screening was performed using allele specific oligonucleotide polymerase chain reaction (ASO-PCR) methods previously described [20]. Bcr/abl mutations that are reported to be associated with imatinib mesylate resistance have all been screened [21].

### Detection of AQP5 gene amplification

FISH of the bone marrow cells was performed in 41 CML patients and 3 control cases. The probe for AQP5 was derived from Homo sapiens 12 BAC RP11-469H8 containing the whole AQP5 gene (GenBank Accession no. AC025154), and labeled with Cy3 (Macrogen, Seoul, Korea). Two genomic DNA clones franking 12p13.33 region labeled with FITC were used as control probes (Macrogen). FISH was performed according to the manufacturer's instructions.

### Statistical Analysis

Fisher-Freeman-Halton exact test and Student's t-test were used to assess the association between AQP5 expression and demographic and clinicopathological variables. In addition, Wilcoxon Signed-Rank test was used to compare the AQP5 expression level before and after the emergence of imatinib mesylate resistance. Differences found between samples in cell proliferation assay, cell counting, and apoptosis assay were examined by Student's t-test. All p-values were derived from two-sided test and were considered to be statistically significant if less than 0.05. All statistical analyses were performed using STATA Statistical Software, 9.0 (Stata Corporation, College Station, TX, USA).

## Results

### AQP5 is expressed in human CML cells

First, we studied the expression profile of AQP5 in leukemic cell lines as well as CML primary cells. With RT-PCR analysis, K562, EM-2, and LAMA-84 human CML cell lines and U937 human monoblastic leukemia cell line showed a clear expression of AQP5 (Figure 1A). This finding was confirmed with mRNA quantification in primary CML cells (Figure 1B). Standardized mRNA levels of AQP5 (AQP5 mRNA/GAPDH mRNA) in lung and breast cancer tissue (positive control) and normal peripheral blood lymphocytes (negative control) were compared to that of CML cells for comparison (Figure 1B). The expression level of AQP5 in CML cells was lower than that of lung and breast cancer tissues. However, AQP5 expression was rarely detected in normal peripheral blood lymphocytes. Then, the protein expression level of AQP5 was examined with bone marrow biopsy samples by immunohistochemisty (Table 1, Table S1). While all the normal bone marrow biopsy samples (n = 5) showed no expression of AQP5 (Figure 1C), 13 out of 41 (32%) CML patient samples demonstrated AQP5 expression (Figure 1D). Among those with expression, 10 samples were clearly positive, while 3 samples were trace (Figure 1D). In positive cases of CML, megakaryocytes and myeloblasts showed weak cytoplasmic staining, whereas granulcytes exhibited markedly strong staining (Figure 1D). As expected, the staining intensity of AQP5 in CML cells was weaker than that of lung tumor cells (positive control), consistent with the mRNA findings (Figure 1C) and our recent observations in NSCLC [17], [19]. For bone marrow cells, mRNA level of AQP5 could not be examined owing to the insufficient amount of the samples. However, a direct association between expression level detected from RT-PCR analysis and immunohistochemistry was found in our preliminary analysis. Therefore, it is probable that RT-PCR analysis would demonstrate similar results.

**Table 1:** AQP5 expression and demographic and clinopathologic characteristics of the CML patients.

Patient characteristics	AQP5 expression		p value‡
	−	+	
Sex, Female	53.6% (15/28)	53.8% (7/13)	0.744
Age, mean (SD)	41.6 (13.4)	44.3 (17.7)	0.596
bcr-abl amplification	25.0% (7/28)	15.4% (2/13)	0.692
bcr-abl mutation	25.0% (7/28)	30.8% (4/13)	0.719
Secondary chromosomal changes*	35.7% (10/28)	46.2% (6/13)	0.732
Phase at diagnosis			**0.003**
Chronic	75.0% (21/28)	38.5% (5/13)	
Accelerated	10.7% (3/28)	38.5% (5/13)	
Blast crisis	3.6% (1/28)	7.7% (1/13)	
Imatinib mesylate resistance	50.0% (14/28)	61.5% (8/13)	0.524
Phase at resistance			**0.004**
Chronic	14.3% (2/14)	50.0% (4/8)	
Accelerated	0% (0/14)	25.0% (2/8)	
Blast crisis	71.4% (10/14)	25.0% (2/8)	
Days to resistance†, mean (SD)	559 (351)	636 (320)	0.437

* Secondary chromosomal change denotes abnormal changes detected from conventional karyotyping test other than bcr-abl amplification; t(9,22).

† Time to resistance represents the interval between the days imatinib mesylate was started to the day resistance to the drug emerged.

‡ P values were derived from Student's t-test (age and days to resistance) and Fisher-Freeman-Halton exact (other variables).

SD denotes standard deviation.

### AQP5 expression may be associated with the aggressiveness in CML progression

We then decided to see whether AQP5 expression in CML cells bears any clinical significance. Thus, we gathered demographic and clinopathologic information of the CML patients (Table 1, Table S1) and analyzed their association with AQP5 expression level. The expression level of AQP5 was associated with CML phase at the time of diagnosis and of resistance (p = 0.03, p = 0.04, respectively). Interestingly, AQP5 expression level was relatively higher among CML patients diagnosed at accelerated, or blast crisis, phase than those diagnosed at chronic phase (Table 1). On the other hand, AQP5 expression level was relatively higher among CML patients who gained imatinib mesylate resistance at chronic phase than those who gained resistance at accelerated, or blast crisis, phase. These findings may allude to the notion that AQP5 expression is linked with more aggressive behavior in CML progression.

No statistically significant relationship was found between the expression level of AQP5 and patient's age, sex, the time interval from diagnosis to resistance, and the time interval from resistance to sampling. In general, there was no statistically significant relationship between AQP5 expression level and the existence of imatinib mesylate resistance in the bone marrow samples (n = 41, p = 0.524). However, when AQP5 expression levels were compared between the paired bone marrow samples drawn before and after the emergence of imatinib mesylate resistance in the same CML patients (n = 9), a statistically significant difference was found (p = 0.047) (Table 2). Intriguingly, AQP5 expression increased with the appearance of imatinib mesylate resistance. This finding suggests that AQP5 may play a role in the process of developing imatinib mesylate resistance in CML. Accordingly, we have further examined whether AQP5 expression in CML cells is linked with bcr-abl amplification, or mutation, or secondary chromosomal changes other than t(9,22) in imatinib mesylate resistant CML cases. In our 41 CML bone marrow samples, although a gain of bcr-abl amplification, mutation, and secondary chromosomal changes were found to be associated with the development of imatinib mesylate resistance (p = 0.002, p<0.001, p = 0.001, respectively), none of them showed a statistically significant correlation with AQP5 expression (Table S1). In paired bone marrow samples drawn before and after the emergence of imatinib mesylate resistance, like AQP5 expression, a gain of bcr-abl mutation revealed an independent association with the development of drug resistance (p = 0.025) (Table 2). Thus, AQP5 expression in CML does not seem to be associated with the major mechanisms of imatinib mesylate resistance including bcr-abl amplification and mutation [21].

**Table 2:** AQP5 expression level and CML Patient information.

patient	AQP5 expression		bcr-abl amplification		bcr-abl mutation		secondary chromosomal change*	
	Pre R	Post R	Pre R	Post R	Pre R	Post R	Pre R	Post R
A	−	−	−	+	−	+	−	+
B	−	++	−	−	−	+	−	+
C	++	++	−	−	−	+	+	+
D	−	−	−	−	−	−	+	−
E	−	+	−	−	−	−	−	−
F	−	+	−	−	−	+	−	+
G	−	−	−	−	−	+	−	−
H	−	−	−	−	−	−	−	+
I	+	++	−	−	−	−	−	−

* Secondary chromosomal change denotes abnormal changes detected from conventional karyotyping test other than bcr-abl amplification; t(9,22).

preR and postR denote AQP5 expression level, or the existence of bcr-abl amplification, mutation, or secondary chromosomal change before and after the emergence of imatinib mesylate resistance, respectively.

### AQP5 expression stimulates CML cell proliferation

**Figure 2:**
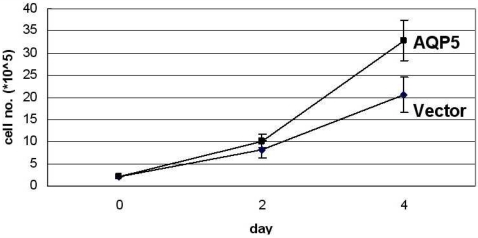
Cell proliferation assay in K562 CML cell line stably expressing AQP5. Cell proliferation was measured with cell counting kit between AQP5 wild-type (WT) and pcDNA vector transfected K562 stable cell lines. The former showed significantly higher proliferation rate than the latter (p<0.01).

Based on our results from the expression assay and the clinical correlation analyses, we chose to explore the molecular mechanism behind the expression of AQP5 in CML cells. First, we decided to investigate whether AQP5 promotes cell proliferation in CML cells by measuring cell proliferation rates between K562 CML cell lines stably transfected with AQP5 and vector pcDNA. Notably, the former showed a significantly higher proliferation rate than the latter (p<0.01) (Figure 2). Also, similar results were replicated in other CML cell lines such as EM-2 and LAMA-84 (data not shown).

### AQP5 activates cell signaling pathway molecules in CML cells

**Figure 3:**
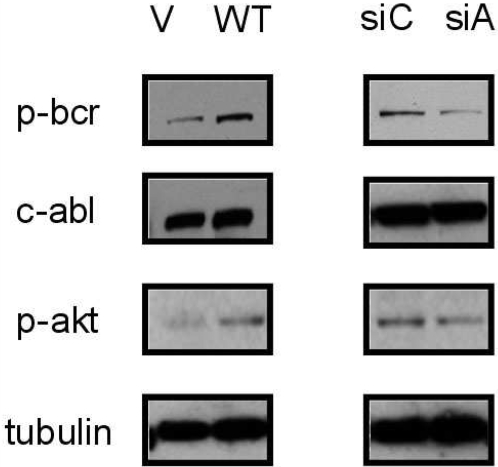
Western blotting analysis in K562 CML cell lines transfected with AQP5 pcDNA and AQP5 siRNA. Western blotting analysis was performed in AQP5 WT versus vector (V) pcDNA transfected K562 stable cell lines and pooled AQP5 siRNA versus pooled control siRNA transfected K562 cell lines. While AQP5 transfected cell line displayed increased BCR (Tyr177) and Akt (Thr308) phosphorylation, AQP5 siRNA transfected cell line showed the opposite. siC, control siRNA treated; siA, AQP5 siRNA treated, c-ABL, and tubulin were loaded as controls.

Then, we checked whether AQP5 expression triggers the activation of cell signaling molecules involved in CML cell proliferation. Of note, K562 CML cells stably overexpressing AQP5 demonstrated an increase in BCR (Tyr177) phosphorylation as well as Akt (Thr308) phosphorylation (Figure 3). Since BCR (BCR-ABL1) and Akt are cell signaling molecules critical for the proliferation of CML cells [22], our finding provides a molecular basis for the involvement of AQP5 in the progression of CML.

### Ablation of AQP5 induces not only decreased cell proliferation but also enhanced apoptosis in CML cells

**Figure 4:**
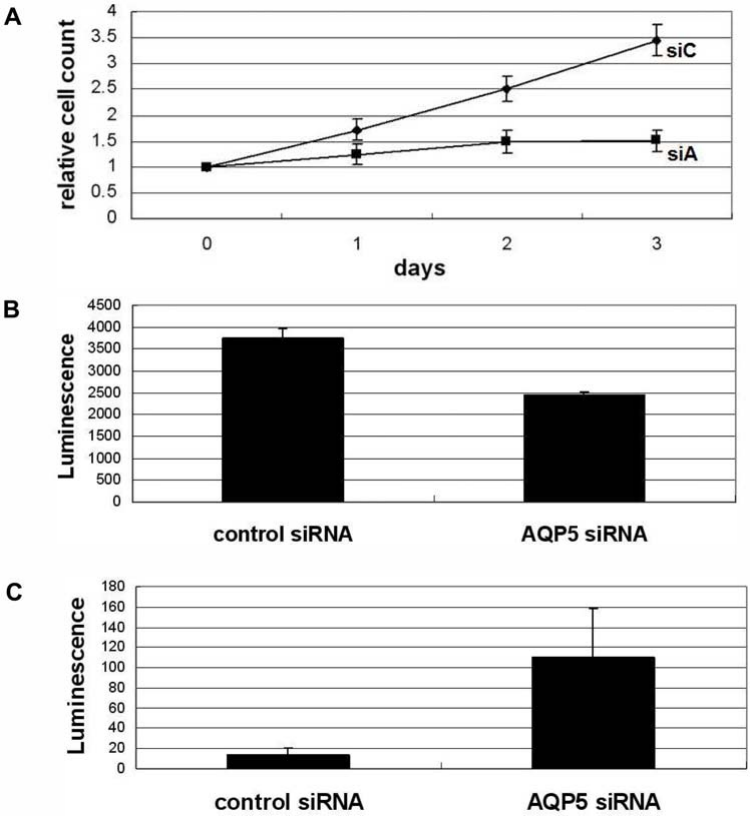
AQP5 small interfering RNA experiment in LAMA-84 CML cell line. (A) Cell counting, (B) ATP concentration and (C) Caspase 9 assay was done (B, C on the 2nd day of transfection) in pooled AQP5 siRNA and pooled control siRNA transfected LAMA-84 cell lines. While AQP5 siRNA treated cell line demonstrated a notably decreased proliferation and cell viability (ATP concentration), it showed a markedly increased caspase 9 activity compared with control siRNA treated cell line (all p values<0.01).

We have decided to validate our findings from AQP5 overexpression study by ablating AQP5 expression with a small inferring RNA (siRNA). As expected, LAMA-84 CML cells treated with AQP5 siRNA showed a marked decrease in cell proliferation compared with cells treated with control siRNA (p<0.01) (Figure 4A). This was further confirmed by examining ATP concentration reflecting viable cell numbers between the two cell lines (Figure 4B); cells treated with control siRNA demonstrated higher number of viable cells than cells treated with AQP5 siRNA (p<0.01). We used LAMA-84 cell line in addition to K562 cell line, since it showed the highest siRNA transfection efficiency among CML cell lines in our preliminary analysis. The ability of the pooled siRNA to silence AQP5 expression was validated with RT-PCR analysis. Similar results were also replicated with K562 cell line (data not shown).

**Figure 5:**
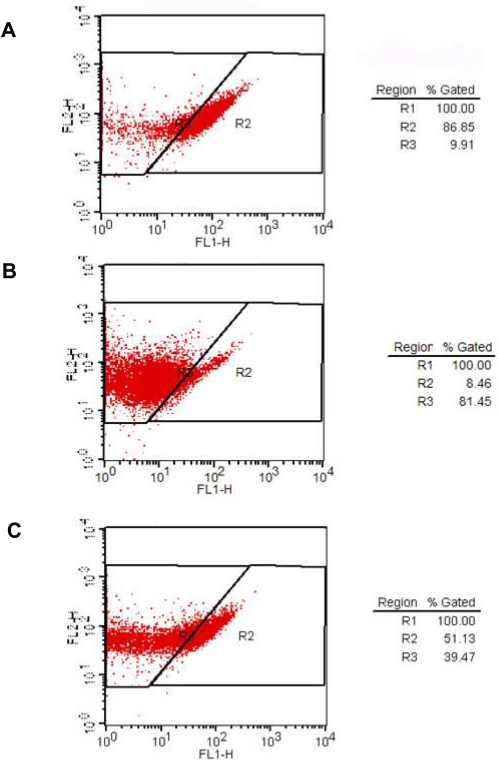
Flow cytometry analysis in AQP5 siRNA treated LAMA-84 CML cell line. Flow cytometry analysis was performed in pooled control siRNA (negative control) (A), imatinib mesylate of 500 nM concentration (positive control) (B) and pooled AQP5 siRNA (C) transfected LAMA-84 cell lines. Cells treated with control siRNA showed a significantly decreased phosphorylation in BCR (Tyr177) than cells treated with AQP5 siRNA. Cells treated with imatinib mesylate showed better inhibition of BCR phosphorylation than cells treated with AQP5 siRNA.

In addition, deactivation of cell signaling molecules was also demonstrated in K562 cells treated with AQP5 siRNA compared with cells treated with control siRNA (Figure 3); the former showed decreased phosphorylation of BCR (Tyr177) and Akt (Thr308) than the latter. We also validated this finding with LAMA-84 cell line using flow cytometry analysis (Figure 5). Imatinib mesylate treatment was used as a positive control in terms of deactivating BCR-ABL1 tyrosine kinase. As expected, cells transfected with AQP5 siRNA demonstrated decreased phosphorylation of BCR (Tyr177), reflecting deactivation of BCR-ABL1, compared with cells transfected with control siRNA (Figure 5A, 5C). However, cells treated with imatinib mesylate showed a stronger inhibition of BCR-ABL1 than cells treated with AQP5 siRNA (Figure 5A, 5B). These findings combined with the expression study results corroborate our hypothesis that AQP5 expression promotes cell proliferation in CML.

To our surprise, we came across a noteworthy finding that LAMA-84 CML cells transfected with AQP5 siRNA exhibited a significantly higher caspase 9 activity than cells transfected with control siRNA (p<0.01) (Figure 4C). Although not statistically significant, similar results were also shown for caspase 3, downstream pathway molecule of caspase 9 (data not shown). Furthermore, light microscopy and Trypsin Blue staining confirmed the differences in the number of cells undergoing apoptosis; caspase 8 activity showed no difference between two cell lines (data not shown). These findings indicate that AQP5 expression may play a role in inhibiting apoptosis in CML cells, possibly through the caspase 9 pathway.

### AQP5 expression in CML cells is not associated with genomic amplification of AQP5

Finally, to elucidate the molecular mechanisms underlying AQP5 expression in CML cells, we investigated the presence of genomic amplification through fluorescence in situ (FISH) analysis using an AQP5 gene probe. Out of 41 cases of bone marrow aspirate samples of CML patient, no clear pattern of genomic amplification was detected (Figure S2), suggesting that the expression of AQP5 could be a secondary molecular event.

## Discussion

Previous expression studies of AQP5 in human tissues, both normal and malignant, have been only in epithelial tissues and carcinomas. For instance, lung epithelium is a well known site of AQP5 expression [5] and the expression level of AQP5 seems to be higher in non small cell lung cancer than in normal lung tissue [22]. Of interest, it has been reported that aspirates of bone marrow cells, fresh or cultured, can express AQP5, typically expressed in the type I pneumocyte [23], and that bone morrow derived cells can be precursors of differentiated parenchymal cells of the lung [23], [24]. Additionally, our group has previously shown that human lymphocytes, when activated, can express AQP5 [13]. Based on these interesting observations and our findings from human leukemic cell lines (Figure 1A), we have decided to study the expression profile of AQP5 in human bone marrow, both normal and malignant. Among 41 bone marrow biopsy samples from CML patients, 32% exhibited AQP5 expression (Figure 1D). However, normal bone marrow samples (n = 5) showed no expression of AQP5 (Figure 1C). This expression profile in CML is comparable to that in solid tumors; AQP5 is expressed in 35% (144/408) of non-small-cell lung cancer [19], and similar observations were also found in breast cancer (manuscript in preparation). However, the relative mRNA expression level of AQP5 in CML cells is found to be lower than that of lung and breast cancer (Figure 1B). Interestingly, unlike carcinoma cells where AQP5 shows membranous expression (Figure 1C), in the bone marrows of CML patients, megakaryocytes, myeloblasts, and granulocytes demonstrated cytoplasmic expression of AQP5 (Figure 1D). Thus, our findings, for the first time, describe the unique expression pattern of AQP5 in the human bone marrow of CML cases.

More interestingly, we found evidence that AQP5 may be associated with the progression of CML. This finding is also comparable to our previous findings that AQP5 expression correlates with clinical outcomes in solid tumors, such as lung metastasis in colon cancer [18], earlier disease progression in lung cancer [19], and worse survival in breast cancer (manuscript in preparation). CML patients diagnosed at accelerated or blast crisis phase showed significantly higher level of AQP5 expression than those diagnosed at chronic phase, while CML patients who gained imatinib mesylate resistance at chronic phase exhibited significantly higher level of AQP5 expression than those who gained resistance at accelerated or blast crisis phase (p = 0.03, p = 0.04, respectively). Furthermore, with paired sample analysis in the same patients (Table 2), AQP5 expression increased with the appearance of imatinib mesylate resistance (p = 0.047). Based on these observations, we suspect that AQP5 may be conferring a growth advantage in the process of CML progression. Furthermore, from our association study with the cytogenetic response analysis (Table 2), we surmise that AQP5 may play a role in developing imatinib mesylate resistance, irrespective of other known major resistance mechanisms such as bcr-abl mutation or amplification [21]. Further experiments are planned to examine whether AQP5 ablation in combination with imatinib mesylate treatment increases the susceptibility of the latter in imatinib mesylate resistant CML cells.

Of late, the molecular mechanism underlying the observed association between AQPs and human cancers has become the focus of our research. We have previously demonstrated the oncogenic property of AQP1 in NIH3T3 mouse fibroblast cell line^23^ and that of AQP5 in the same cell line [17]. Recently, we have found that AQP5 expression in NIH3T3 cell line induces cell proliferation through the activation of Ras, suggesting an association between AQP5 and the Ras signal transduction pathway [16], [17]. In addition, we have reported that AQP5 triggers Ras/ERK/Rb pathway in HCT116 colon cancer cell line, thereby promoting cell proliferation [18]. Furthermore, we have observed that AQP5 activates c-Src, and, thus, triggers cell invasion and epithelial-mesenchymal transition (EMT) in BEAS-2B human bronchial epithelial cell line [19]. However, these finding were all based on solid tumors and the role of AQPs in the development of hematological malignancies are largely unknown. Thus, in this study, we have pursued the role of AQP5 in CML as a first model to study the role of AQPs in blood cancer.

We report, for the first time, that AQP5 promotes cell proliferation in CML cell lines possibly through the activation of BCR-ABL1 and Akt. We have also found that AQP5 may prolong cell survival by inhibiting apoptosis via caspase 9 pathway. We believe that these findings may shed light in discovering novel molecular targets for CML treatment, where drug resistance has become a huge obstacle for oncologists [21]. However, there needs to be further investigation into several other candidate cell signaling pathways important in CML progression that may be affected by AQP5. Our next plan is to examine changes in the signal transduction molecules related with the BCR-ABL1 pathway such as CrkL, Ras, Jak2, Stat5, and c-Myc [26] as well as molecules that are not linked with BCR-ABL1 pathway including Lyn [30] in both AQP5-overexpressing and AQP5-silenced CML cell lines. Also, we plan to delve into the details of the apoptosis pathways including Bcl-2, Bax, Bad, and Fas [27]. Ultimately, our findings including proliferation assay and molecular expression assays await further validation with primary CML cells.

One question that may arise is the identification of the adaptor molecule that directly interacts with AQP5 to exert its alleged effects in CML. One possibility is for AQP5 to interact with and, thus, activate Lyn in CML cells. With protein microarray and GST pull-down assay, we have reported that AQP5 binds to Lyn [19], the activation of which has been found to be one of the mechanisms of imatinib mesylate resistance [28]. Similar to our findings, Lyn ablation led to enhanced apoptosis of CML cells, even in primary cells resistant to imatinib mesylate therapy [29]. Although the pathway of Lyn activation seems to be independent of BCR-ABL1 [28], it would be worthwhile to explore the possible interaction between Lyn and AQP5 in CML cells and see how AQP5 ablation affects the growth of imatinib mesylate-resistant primary CML cells. In addition, our recent studies suggest that Grb2 (manuscript submitted) and c-Src [19], both of which showed *in vitro* and *in vivo* binding with AQP5, could be some of the candidate adaptor molecules which interact with AQP5 in CML cells. Thus, we plan on performing binding assays with the above candidate molecules in CML cell lines and primary CML cells. Lastly, the possibility that AQP5 can function as a kinase itself should not be ignored.

Another question that remains is why AQP5 is overexpressed in certain CML cells. In our FISH analysis (n = 41), we found no genomic amplification of AQP5 (Figure S2), indicating that AQP5 expression may be a secondary phenomenon. This is similar to our finding with AQP1 [25] and AQP5 [19] in lung, where no evidence for genomic amplification was detected. Currently, we are investigating the presence of AQP5 mutation with the CML samples, especially with the paired samples drawn before and after the emergence of imatinib mesylate resistance in the same CML patients. Our previous efforts to identify a promoter element responsible for the induced expression of AQP5 in several tumor cell lines suggested the existence of several potential cis-acting elements responsible for promoter activity. Moreover, methylation analysis of the AQP5 promoter region suggested that promoter demethylation may play a role in the expression of AQP5 in head and neck and lung cancer cell lines (manuscript in preparation). These results, however, are still preliminary and warrant further validation.

In conclusion, this is the first report, to our knowledge, to show that human AQP5 is expressed in CML cells with a potential role in cancer progression and plays a role not only in proliferation, but also in survival of CML cells. Moreover, they also provide a potential basis for designing a novel CML therapy targeting AQP5. Further expression profile studies in other hematologic malignancies such as acute leukemia and lymphoma are planned. Comprehensive functional studies to verify the novel oncogenic and anti-apoptotic properties of AQP5 in blood cancer are also warranted.

## Supporting Information

Methods S1 (0.03 MB DOC)

Table S1. Table S1. Demographic and clinocopathologic characteristics of the CML patients.
(0.11 MB DOC)

Figure S1 Transfection effieciency in K562 cell line. eGFP (enhanced green fluorescence proteins) transfected into K562 CML cell lines show green fluorescence in fluorescence microscopy (left side) versus no fluorescence in light microscopy (right side). Estimated transfection efficiency using Nucleofactor (Amaxa) was 60–70 percent as shown above.
(15.30 MB DOC)

Figure S2 Figure 5. FISH analysis in CML cells. FISH analysis in a CML case is showing no genomic amplification of AQP5 (green color: FITC labeled control probe, red color: rhodamin labeled AQP5 probe). Original magnification ×1000.
(0.15 MB DOC)
