# Supplementary material for: Human AQP5 Plays a Role in the Progression of Chronic Myelogenous Leukemia (CML)
Source: PLoS One. 2008 Jul 9;3(7):e2594. doi: 10.1371/journal.pone.0002594 (PMC2440422; doi:10.1371/journal.pone.0002594)
Supplement: Methods S1 — (0.03 MB DOC) [file pone.0002594.s001.doc]

**Supplementary Methods**

Four siRNAs targeting human AQP5 and four nonspecific siRNAs as a negative control each combined into one pool were designed and synthesized from Dharmacon (CO, USA).

**siRNA sequences provided form Dharmacon**

**SMARTpool:**

Gene name: Human Aquaporin 5 (AQP5)

Accession number: NM_001651

Catalog number: L-004520-00-0005

Full Sequence

Duplex 1: GCUCCGGGCUUUCUUCUACUU

Duplex 2: GAACCCAGCCCGCUCUUUUUU

Duplex 3: CGUAUGAGCCUGACGAGGAUU

Duplex 4: GCGCUCAACAACAACACAAUU

Catalog number: D-001206-13-05

Product description: nonspecific control duplexes

Accession number: none

Full Sequence

Duplex 1: AUGAACGUGAAUUGCUCAA

Duplex 2: UAAGGCUAUGAAGAGAUAC

Duplex 3: AUGUAUUGGCCUGUAUUAG

Duplex 4: UAGCGACUAAACACAUCAA
