# Supplementary material for: Human AQP5 Plays a Role in the Progression of Chronic Myelogenous Leukemia (CML)
Source: PLoS One. 2008 Jul 9;3(7):e2594. doi: 10.1371/journal.pone.0002594 (PMC2440422; doi:10.1371/journal.pone.0002594)
Supplement: Table S1. — Demographic and clinocopathologic characteristics of the CML patients. (0.11 MB DOC) [file pone.0002594.s002.doc]

**Table S1. Demographic and clinocopathologic characteristics of the CML patients.**

| **Patient no.** | **Sex** | **Age** | **AQP5** | **bcr-abl amplification** | **bcr-abl mutation** | **Secondary chromosomal change*** | **Phase at diagnosis** | **Imatinib resistance** | **Phase at resistance** | **Days to resistance**† |
| --- | --- | --- | --- | --- | --- | --- | --- | --- | --- | --- |
| 1 | M | 25 | - | - | - | - | accelerated | - |  |  |
| 2 | M | 60 | - | - | - | - | accelerated | - |  |  |
| 3 | F | 33 | - | - | - | - | accelerated | - |  |  |
| 4 | M | 25 | - | - | - | - | chronic | - |  |  |
| 5 | F | 22 | - | - | - | - | chronic | - |  |  |
| 6 | F | 58 | - | - | - | - | chronic | - |  |  |
| 7 | F | 43 | - | - | - | - | NA | - |  |  |
| 8 | F | 37 | - | - | - | - | chronic | - |  |  |
| 9 | F | 48 | - | - | - | - | chronic | - |  |  |
| 10 | M | 34 | - | - | - | + | chronic | - |  |  |
| 11 | M | 62 | - | - | - | - | chronic | - |  |  |
| 12 | M | 40 | - | - | - | - | chronic | - |  |  |
| 13 | M | 27 | - | - | - | - | chronic | - |  |  |
| 14 | F | 55 | - | - | - | - | chronic | - |  |  |
| 15 | F | 45 | + | - | - | - | chronic | - |  |  |
| 16 | M | 20 | + | - | - | - | accelerated | - |  |  |
| 17 | M | 42 | ++ | - | - | - | NA | - |  |  |
| 18 | F | 40 | ++ | - | - | - | NA | - |  |  |
| 19 | F | 72 | ++ | - | - | + | accelerated | - |  |  |
| 20 | F | 19 | - | + | + | + | chronic | + | blast crisis | 421 |
| 21 | M | 48 | - | - | + | - | chronic | + | blast crisis | 317 |
| 22 | F | 37 | - | + | + | + | chronic | + | blast crisis | 219 |
| 23 | M | 40 | - | + | - | + | chronic | + | blast crisis | 372 |
| 24 | F | 56 | - | - | - | - | chronic | + | blast crisis | 994 |
| 25 | M | 60 | - | + | - | + | chronic | + | blast crisis | 585 |
| 26 | M | 49 | - | - | - | - | chronic | + | chronic | 655 |
| 27 | F | 33 | - | - | + | + | NA | + | NA | NA |
| 28 | F | 64 | - | + | + | + | NA | + | NA | NA |
| 29 | F | 31 | - | - | - | + | blast crisis | + | blast crisis | NA |
| 30 | F | 37 | - | + | + | + | chronic | + | blast crisis | 236 |
| 31 | M | 37 | - | - | - | - | chronic | + | chronic | 1002 |
| 32 | M | 29 | - | - | + | - | chronic | + | blast crisis | 183 |
| 33 | F | 57 | - | + | - | + | chronic | + | blast crisis | 1162 |
| 34 | M | 63 | + | - | - | - | chronic | + | chronic | 775 |
| 35 | M | 40 | + | - | + | + | chronic | + | blast crisis | 166 |
| 36 | M | 25 | ++ | - | + | - | chronic | + | chronic | 411 |
| 37 | F | 54 | ++ | - | - | + | accelerated | + | accelerated | NA |
| 38 | M | 32 | ++ | + | - | + | blast crisis | + | blast crisis | NA |
| 39 | F | 49 | ++ | - | + | + | chronic | + | accelerated | 863 |
| 40 | F | 73 | ++ | - | + | + | accelerated | + | chronic | 657 |
| 41 | F | 21 | ++ | + | - | - | accelerated | + | chronic | 1049 |

*Secondary chromosomal change denotes abnormal changes detected from conventional karyotyping test other than bcr-abl amplification; t(9,22).

† Time to resistance represents the interval between the days imatinib mesylate was started to the day resistance to the drug emerged.

M, F, and NA denote male, female, and data not available.
