# Supplementary figures and images for: Human AQP5 Plays a Role in the Progression of Chronic Myelogenous Leukemia (CML)
Source: PLoS One. 2008 Jul 9;3(7):e2594. doi: 10.1371/journal.pone.0002594 (PMC2440422; doi:10.1371/journal.pone.0002594)

**Figure S1.**


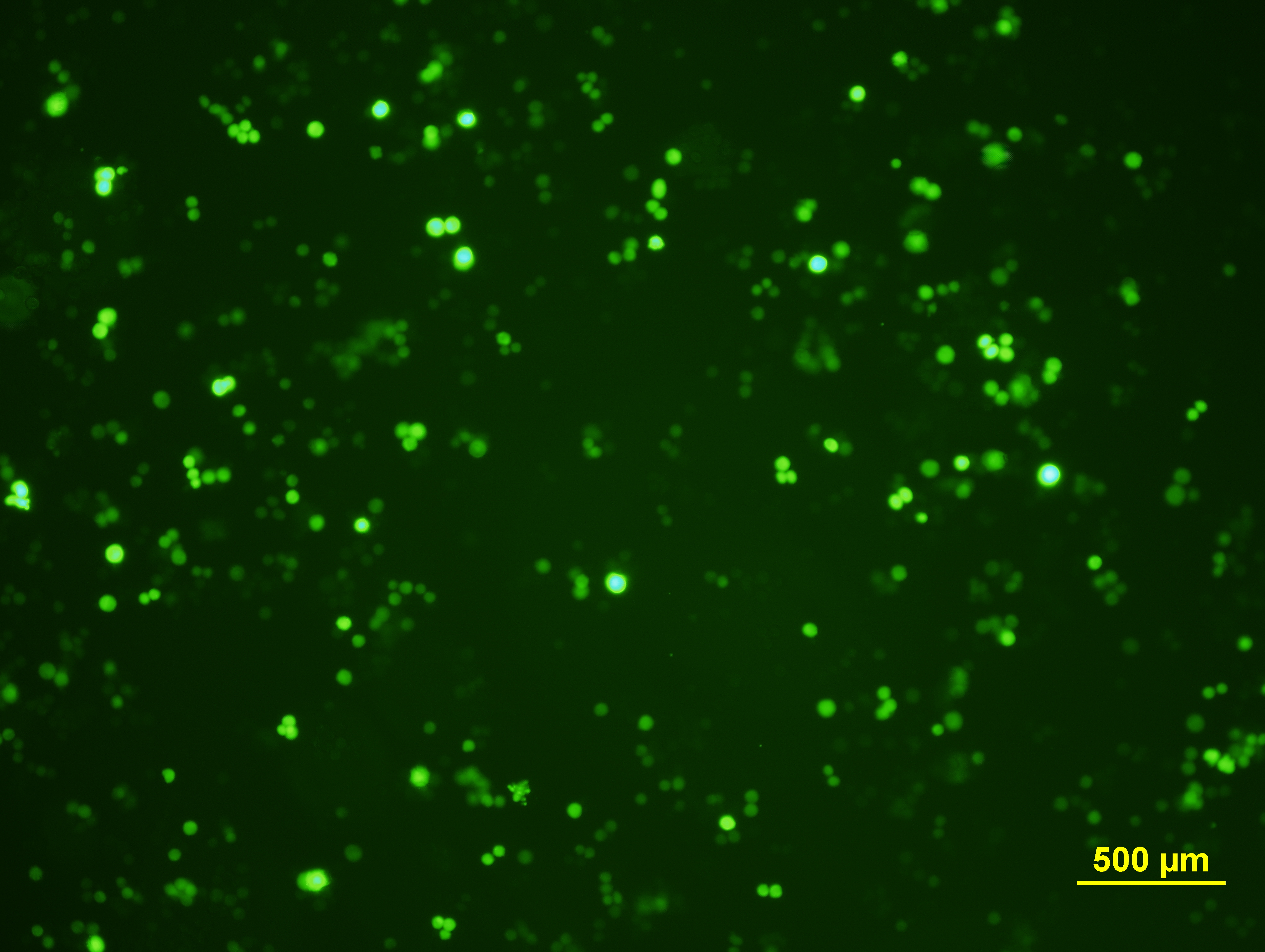

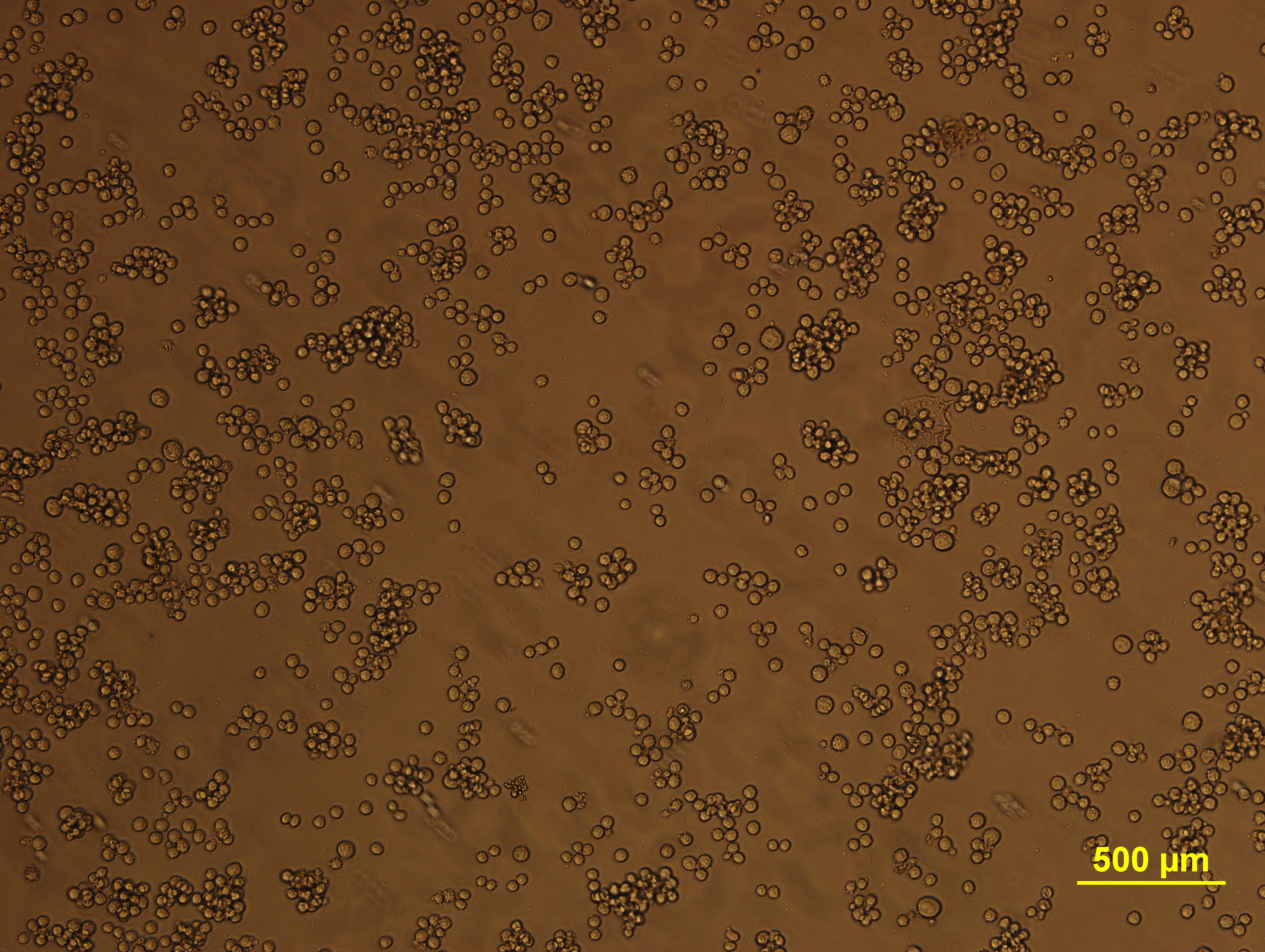

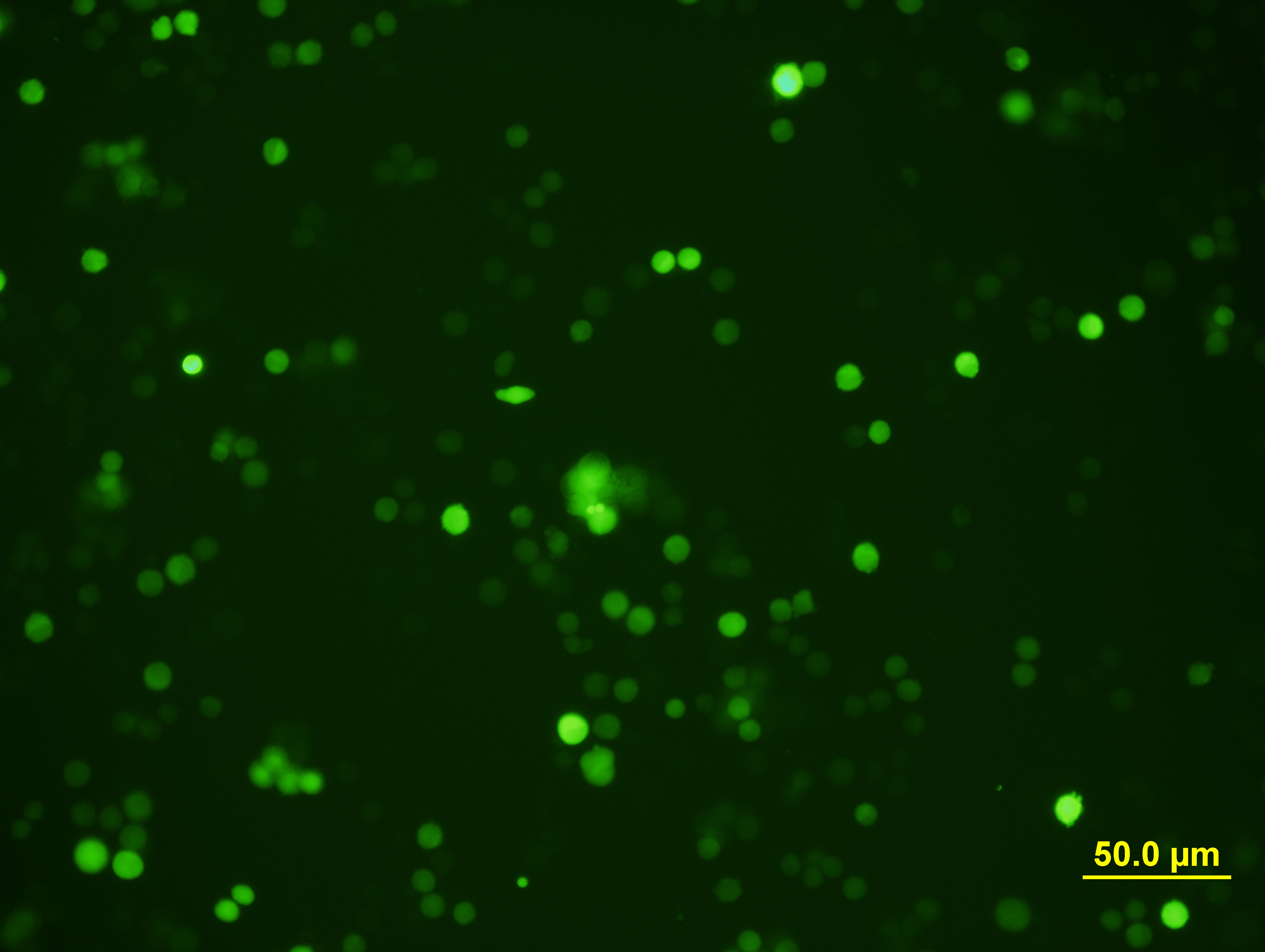

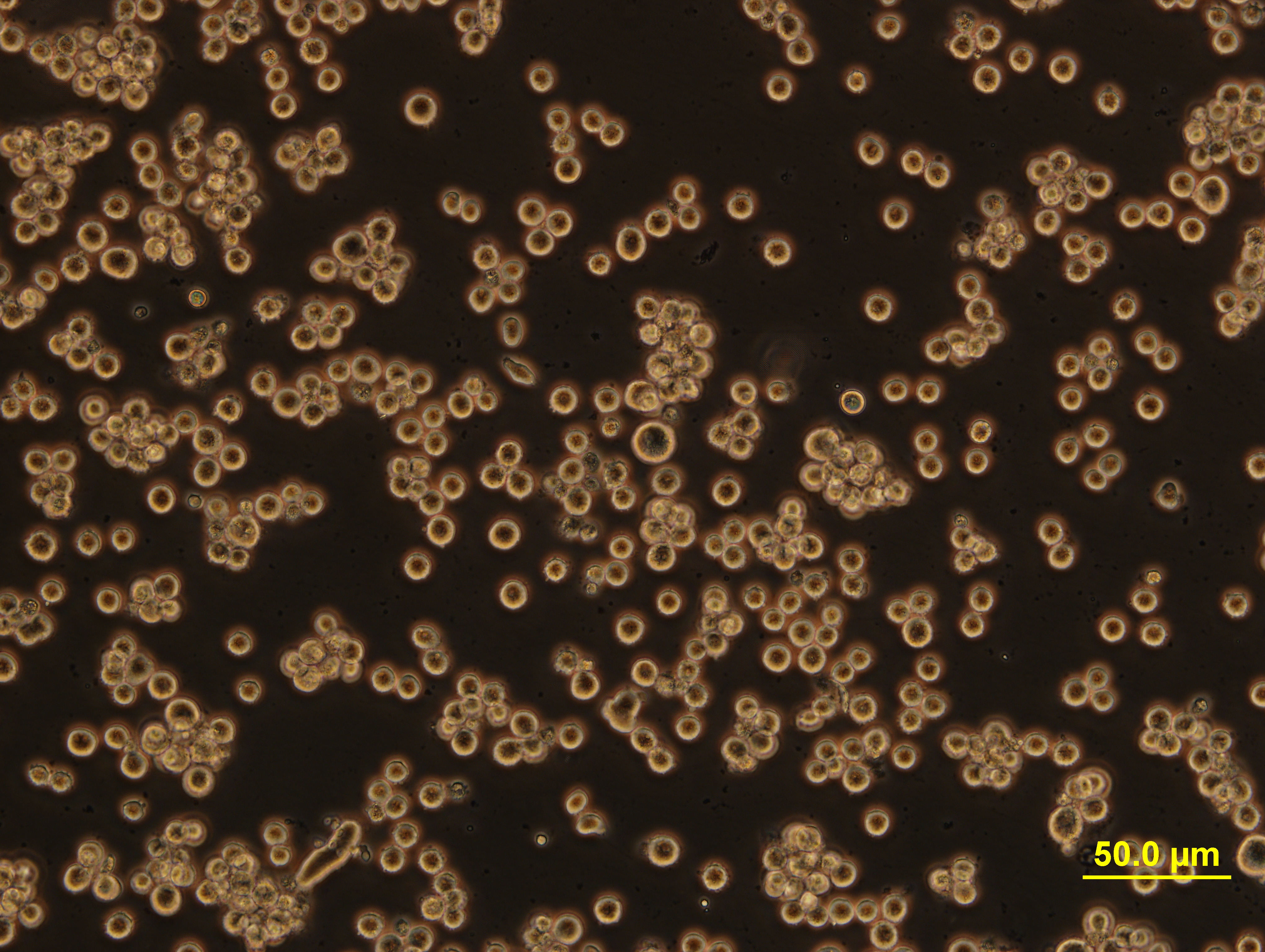

Supplement: Figure S1 — Transfection effieciency in K562 cell line. eGFP (enhanced green fluorescence proteins) transfected into K562 CML cell lines show green fluorescence in fluorescence microscopy (left side) versus no fluorescence in light microscopy (right side). Estimated transfection efficiency using Nucleofactor (Amaxa) was 60–70 percent as shown above. (15.30 MB DOC) [file pone.0002594.s003.doc]

**Figure S2.**


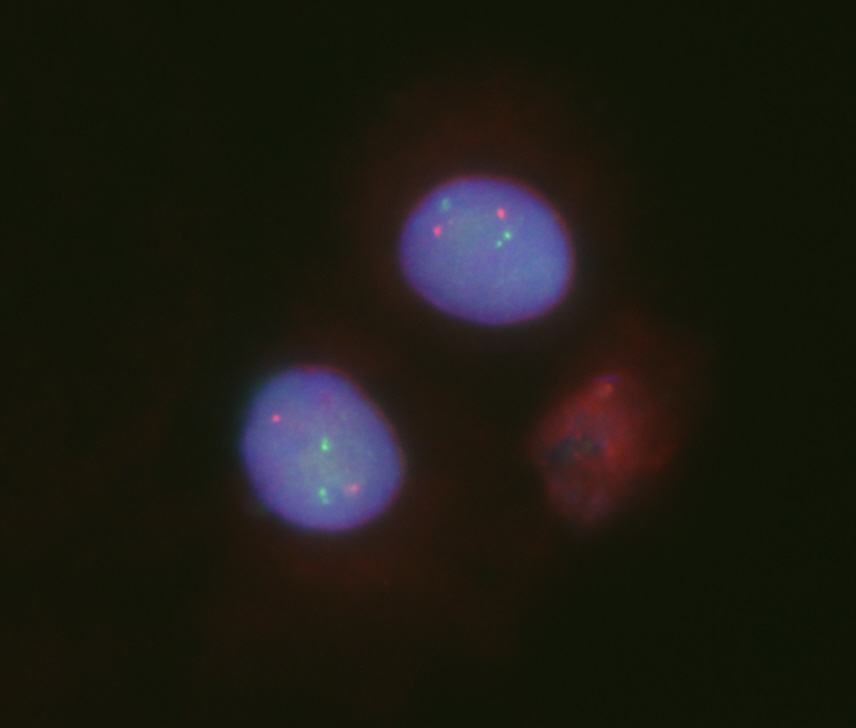

Supplement: Figure S2 — Figure 5. FISH analysis in CML cells. FISH analysis in a CML case is showing no genomic amplification of AQP5 (green color: FITC labeled control probe, red color: rhodamin labeled AQP5 probe). Original magnification ×1000. (0.15 MB DOC) [file pone.0002594.s004.doc]
